# Impact of Tele-Emergency Consultations on Pediatric Interfacility Transfers: A Cluster-Randomized Crossover Trial

**DOI:** 10.1001/jamanetworkopen.2022.55770

**Published:** 2023-02-13

**Authors:** James P. Marcin, Hadley S. Sauers-Ford, Jamie L. Mouzoon, Sarah C. Haynes, Parul Dayal, Ilana Sigal, Daniel Tancredi, Monica K. Lieng, Nathan Kuppermann

**Affiliations:** 1 Department of Pediatrics, University of California, Davis School of Medicine, Sacramento; 2 Genentech Inc, South San Francisco, California; 3 Department of Emergency Medicine, University of California, Davis School of Medicine, Sacramento

## Abstract

**Importance:**

Pediatric referral centers are increasingly using telemedicine to provide consultations to help care for acutely ill children presenting to rural and community emergency departments (EDs). These pediatric telemedicine consultations may help improve physician decision-making and may reduce the frequency of overtriage and interfacility transfers.

**Objective:**

To examine the use of pediatric critical care telemedicine vs telephone consultations associated with risk-adjusted transfer rates of acutely ill children from community and rural EDs.

**Design, Setting, and Participants:**

A cluster-randomized crossover trial was conducted between November 18, 2015, and March 26, 2018. Analyses were conducted from January 19, 2018, to July 23, 2022, 2022. Participants included acutely ill children aged 14 years and younger presenting to a participating ED in 15 rural and community EDs in northern California.

**Interventions:**

Participating EDs were randomized to use telemedicine or telephone for consultations with pediatric critical care physicians according to 1 of 4 unbalanced (3 telemedicine:1 telephone) crossover treatment assignment sequences.

**Main Outcomes and Measures:**

Intention-to-treat, treatment-received, and per-protocol analyses were performed to determine the risk of transfer using mixed effects Poisson regression analyses with random intercepts for presenting EDs to account for hospital-level clustering.

**Results:**

A total of 696 children (392 boys [56.3%]; mean [SD] age, 4.2 [4.6] years) were enrolled. Of the 537 children (77.2%) assigned to telemedicine, 251 (46.7%) received the intervention. In the intention-to-treat analysis, patients assigned to the telemedicine arm were less likely to be transferred compared with patients assigned to the telephone arm after adjusting for patient age, severity of illness, and hospital study period (risk rate [RR], 0.93; 95% CI, 0.88-0.99). The adjusted risk of transfer was significantly lower in the telemedicine arm compared with the telephone arm in both the treatment-received analysis (RR, 0.81; 95% CI, 0.71-0.94) and the per-protocol analysis (RR, 0.79; 95% CI, 0.68-0.92).

**Conclusions and Relevance:**

In this randomized trial, the use of telemedicine to conduct consultations for acutely ill children in rural and community EDs resulted in less frequent overall interfacility transfers than consultations done by telephone.

**Trial Registration:**

ClinicalTrials.gov Identifier: NCT02877810

## Key Points

**Question:**

Does the use of telemedicine for pediatric consultations for acutely ill children to referring hospital emergency departments result in fewer interfacility transfers than the current standard of care, telephone consultations?

**Findings:**

In this cluster-randomized crossover trial that included 696 acutely ill children presenting to 15 community and rural emergency departments, the adjusted risk of transfer was significantly lower by 7% among those assigned to the telemedicine arm than those assigned to the telephone arm. The number-needed-to-treat to prevent 1 transfer was 16.5.

**Meaning:**

The use of telemedicine to conduct consultations for acutely ill children in rural and community emergency departments results in less frequent interfacility transfers than consultations done by telephone.

## Teaser

This cluster randomized trial examines the use of telemedicine by pediatric referral centers to reduce the frequency of interfacility transfers among acutely ill children presenting to rural and community emergency departments.

## Introduction

Emergency departments (EDs) with low pediatric patient volumes and limited access to pediatric clinicians are at risk of delivering suboptimal care, including delays in diagnoses, inappropriate therapies, and noncurrent medical management.^1,2,3^ Because of this risk, and to enhance safety, acutely ill children presenting to EDs without comprehensive pediatric services are often transferred to regional pediatric centers. Many interfacility transfers of children, however, are unnecessary, and often place a large burden on patients and families.^4,5,6^

Telemedicine may be an effective way to provide rural and community EDs with pediatric subspecialty support. More than one-half of EDs in the US use telemedicine for specialty consultations, including mental health, stroke, and other pediatric services.^7,8^ Telemedicine consultations can bring pediatric specialists virtually into EDs to assist in the care of acutely ill children, potentially reducing unnecessary transfers by allowing the pediatric specialist to visually assess the patient and make precise recommendations.^9,10^ While our group and others have shown associations between the use of telemedicine with parent and clinician satisfaction, measures of quality of care, and disposition and transfer decisions in the ED,^9,10,11,12^ to our knowledge, no randomized trials exist.^13,14^ We sought to conduct a pragmatic assessment,^15,16^ designed to evaluate the impact of telemedicine consultations on transfer rates compared with telephone consultations in a robust clinical trial. We hypothesized that the use of telemedicine for pediatric consultations in the ED would result in fewer transfers to a pediatric referral hospital than the use of telephone consultations, the current standard of care.

## Methods

We conducted a pragmatic, cluster-randomized, crossover trial to evaluate the impact of telemedicine consultations on risk-adjusted transfer rates among children presenting to rural and community EDs in northern California. The primary end point for the trial was whether a patient was transferred to the University of California Davis Children’s Hospital (UCDCH) following a pediatric critical care consultation. Pediatric critical care physicians at the UCDCH are available 24 hours daily to provide emergency and critical care telephone and telemedicine consultations to clinicians caring for children receiving care in referring EDs, for both patient management and to facilitate interfacility transfers if indicated. From the more than 30 hospital EDs in the UCDCH catchment area with telemedicine capabilities, we selected 16 EDs to ensure a diverse sample of critical access hospitals (n = 6) including those located in Health Resources and Services Administration–designated rural geographic areas (n = 12). One of the participating hospitals closed early in the trial after conducting only 4 consultations (2 telemedicine and 2 telephone) and because medical records were not available, these patients were not included in the analyses (Figure). We received approval to conduct the study and collect deidentified patient data from the human subjects review committees and/or compliance offices of all participating sites. The study was found exempt from the requirement to obtain informed consent from patients because of minimal risk to participants. We followed the Consolidated Standards of Reporting Trials (CONSORT) reporting guideline for randomized clinical trials (Figure). The original trial protocol is provided in Supplement 1.

**Figure.**
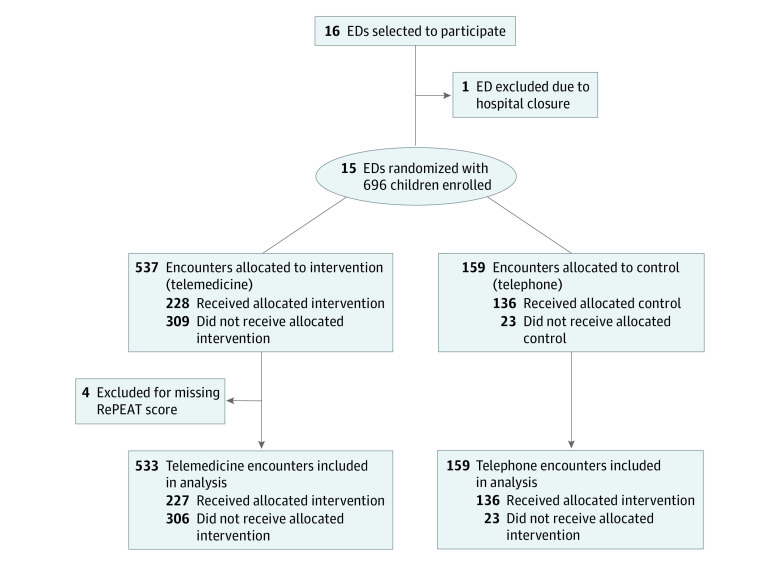
Participant Flow Diagram ED indicates emergency department; RePEAT, Pediatric Emergency Assessment Tool.

### Trial Design and Randomization

Randomization and treatment assignment followed an unbalanced, cluster-randomized crossover design with staggered start dates from November 18, 2015, to March 26, 2018. The unit of randomization was the ED and the treatment allocated was the modality of consultation (T for telemedicine or P for telephone). Participating EDs were randomized to 1 of the 4 unbalanced (3:1) crossover treatment assignment sequences, each consisting of a 6-month period for 2 years: PTTT, TPTT, TTPT, or TTTP. We chose an unbalanced design for this pragmatic trial because we anticipated that telemedicine would not be used in all assigned circumstances as well as the expectation of an intracluster (ED) correlation of 2% or greater.

Prior to trial initiation, there was a 6-month study ramp-up period during which protocol and telemedicine training was conducted at all participating EDs. Subsequently, at the start of the trial, participating sites were randomly allocated the consultation modality for each subsequent 6-month period. During this period, the UCDCH pediatric critical care physicians providing the consultations were made aware of the ED allocation assignments in several ways. First, stickers with ED assignments were placed on the cards used by physicians to document consultations; second, emails were sent to all physicians with every change in ED allocation; and third, signs were displayed at all pediatric critical care physician workstations at the UCDCH. Deviating from protocol (ie, using telephone consultation when randomized to telemedicine or vice versa) could occur at the discretion of the referring and/or consulting physician. Research staff, participating physicians, patients, and their families were not blinded to treatment assignment.

### Participants

Telephone and telemedicine consultations were similarly initiated with the referring ED physician calling the UCDCH transfer center, when they would request a consultation with a pediatric critical care physician, given the patient’s perceived severity of illness and/or need for pediatric intensive care services. We included all children aged 14 years or younger who presented to a participating ED with an acute medical condition that resulted in a consultation with a UCDCH pediatric critical care physician. We did not include children with acute physical trauma because these consultations are directed to on-call pediatric trauma specialists.

### Telemedicine Protocol

Telemedicine consultations involved synchronous audio-video communication and were initiated after the referring ED physician and the UCDCH pediatric critical care physician were connected for a brief telephone conversation via the transfer center. The telemedicine consultation involved the referring physician, bedside nurse, respiratory therapist, patient, and patient’s parents/guardians, when available, and always involved a visual assessment and physical examination of the patient. Telemedicine units in the referring EDs consisted of a pole-mounted, high-resolution videoconferencing unit with pan-tilt-zoom capabilities that use the internet for high-definition video (minimum 1 megabits per second, 720 pixels). During the study period, remote access to the participating ED medical records was not available. Therefore, all components of the medical history, including laboratory test results, were obtained via communication with the referring physician. Telemedicine capabilities at the UCDCH were accessible via workstation computers using videoconferencing software, a headset, and a webcam.

### Data Collection and Variables

We collected UCDCH transfer center reports on all patients who received any consultation during the study. We then used detailed data on consultation requests from participating EDs and clinician notes in the UCDCH electronic medical record to confirm qualifying patient encounters during the study period. Deidentified medical records of qualifying patients were obtained from participating hospitals through secure electronic communications. These were scanned and uploaded into a secure, encrypted, password-protected research database (REDCap).^17,18^

We abstracted demographic variables (age, sex, race and ethnicity, and insurance status), consultation type (telephone, telemedicine), mode of arrival to presenting ED, disposition from presenting ED (discharged, admitted, transferred), mode of transfer (air or ground ambulance), distance of presenting ED from the UCDCH, presenting chief signs and symptoms, presenting encounter diagnoses, and physiologic data. Chief signs and symptoms were categorized into Pediatric Emergency Reason for Visit Clusters.^19^ Race and ethnicity were derived from medical records with categories determined by NIH Racial and Ethnic Categories and Definitions^20^ and then combined into a single variable with 4 distinct options: Hispanic of all races, non-Hispanic White, non-Hispanic Black, and non-Hispanic mixed and unreported races. Mixed race includes American Indian or Alaska Native, Asian, Native Hawaiian or other Pacific Islander, and other race or ethnicity. We computed the Pediatric Emergency Assessment Tool, second generation (RePEAT) score for each patient; this illness severity score includes chief signs and symptoms, age, triage acuity, arrival by emergency medical services, current use of prescription medications, and triage vital signs.^21^ Insurance status was dichotomized into private (commercial employer-based) and nonprivate, which included public insurance (eg, Medicaid and no insurance/self-pay).

### Sample Size

From prior experience, we expected approximately 2% of children presenting to participating EDs to have a pediatric specialty consultation, and of these, 25% to receive a pediatric critical care consultation.^9^ Using conservative estimates, we planned to enroll a minimum of 448 children to achieve at least 80% power to detect a clinically important difference of approximately 14 percentage points in the probability of transfer, assuming a baseline transfer probability of 85%. We conducted these calculations using 2-sided testing with a type I error rate of 5%, after determining via simulation studies that design effects from our cluster randomized approach and analytic strategy could reduce our effective sample size by 32%, compared with a balanced allocation individually randomized design.

### Statistical Analysis

All analyses were conducted in 2022 and included intention-to-treat, treatment-received, and per-protocol analyses (J.P.M, S.C.H., D.T., M.K.L.) in Stata/SE, version 17 (StataCorp LLC). Univariable and bivariable comparisons were conducted using Pearson χ^2^ tests for categorical variables or Wald tests for continuous variables adjusted for clustering at the hospital level using the Stata survey data analysis commands. While some participating hospitals can admit children with simple conditions, the UCDCH is the only pediatric referral center in the area with a pediatric ED, designated pediatric inpatient beds, and pediatric intensive care unit, and is the destination for children with the highest acuity conditions in the region. Mixed-effects Poisson regression models were adjusted for patient age at encounter, the natural logarithm of severity of illness as measured by the RePEAT score, and a 4-level study period variable (in 6-month intervals for each ED) to adjust for potential changes in transfer practices during the duration of the study. Random ED intercepts were used to account for clustering effects and robust SEs to correct for the use of a dichotomous outcome.^22^ Numbers needed to treat to avoid 1 transfer to UCDCH were computed by taking the reciprocal of the adjusted proportion difference based on arm-specific marginal estimates from the Stata postestimation margins command. The inclusion of these variables in the model followed our original study protocol analytic plan.

As a pragmatic trial, we evaluated the distribution of baseline characteristics between encounters that used the assigned modality (telephone vs telemedicine) to identify differences in the study arms. To account for possible carryover effects of telephone assignment in the subsequent telemedicine study period, we included a cluster-varying binary indicator for switch period in the multivariable logistic regression models. We then used a nested likelihood ratio test to compare models with and without this term to formally test for the presence of effect modification by potential carryover effects. The level at which statistical significance was set was 2-sided *P* < .05.

## Results

A total of 696 children (392 [56.3%] boys, 304 [43.7%] girls) with an overall mean (SD) age of 4.2 (4.6) years were enrolled from the 15 participating EDs (Figure). As reported in Table 1, 537 patients (77.2%) were assigned to consultations by telemedicine and 159 (22.8%) were assigned to consultations by telephone. In total, 251 patients (36.1%) received telemedicine consultations and 445 (63.9%) received telephone consultations (treatment-received analysis) (Table 1). Of the patients allocated to telemedicine, 228 (42.5%) received telemedicine consultations (per-protocol analysis) (Table 1). Of the patients allocated to telephone consultations, 136 (85.5%) received telephone consultations and 23 (14.5%) received telemedicine consultations. In the intention-to-treat analysis, we found that patients had similar mean (SD) ages: 4.4 (4.7) years in the telemedicine arm and 3.7 (4.4) years in the telephone arm. Furthermore, there was a similar percentage of girls (telemedicine, 43.0%; telephone, 45.9%; *P* = .62) and similar clinical characteristics, including chief signs and symptoms categories and mean RePEAT probabilities of admissions. There was a lower proportion of patients with private insurance allocated to the telemedicine treatment arm (19.6%) than the telephone arm (24.5%) (*P* = .02) (Table 1).

**Table 1.** Baseline Characteristics of Patients in the Telemedicine and Telephone Arms

Patient factor	Intention-to-treat analysis			Treatment-received analysis			Per-protocol analysis		
	Assigned, No. (%)		*P* value^a^	Received, No. (%)		*P* value^a^	Assigned and received, No. (%		*P* value^a^
	Telephone (n = 159)	Telemedicine (n = 537)		Telephone (n = 445	Telemedicine (n = 251		Telephone (n = 136)	Telemedicine (n = 228)	
Patient age, y									
Mean (SD)	3.67 (4.40)	4.39 (4.67)	.18	4.22 (4.77)	4.22 (4.36)	>.99	3.76 (4.53)	4.33 (4.42)	.42
Median (IQR)	1.63 (0.24-5.59)	2.33 (0.54-7.67)		1.84 (0.31 7.65)	2.39 (0.65-7.43)		1.6 (0.3-6.2)	2.5 (0.7-7.6)	
Sex									
Female	73 (45.9)	231 (43.0)	.62	200 (44.9)	104 (41.4)	.19	61 (44.9)	92 (40.4)	.21
Male	86 (54.1)	306 (57.0)		245 (55.1)	147 (58.6)		75 (55.2)	136 (59.6)	
Insurance status									
Private	39 (24.5)	105 (19.6)	.02	92 (20.7)	52 (20.7)	.99	36 (26.5)	49 (21.5)	.17
Medicaid/self-pay/no insurance/other	120 (75.5)	432 (80.4)		353 (79.3)	199 (79.3)		100 (73.5)	179 (78.5)	
Race and ethnicity									
Hispanic	20 (12.6)	105 (19.6)	.29	76 (17.1)	49 (19.5)	.30	19 (14.0)	48 (21.0)	.37
Non-Hispanic									
Black	13 (8.2)	51 (9.5)		49 (11.0)	15 (6.0)		13 (9.6)	15 (6.6)	
Mixed and unreported races^b^	21 (13.2)	91 (16.9)		66 (14.8)	46 (18.3)		87 (64.0)	123 (53.9)	
White	105 (66.0)	290 (54.0)		254 (57.1)	141 (56.2)		17 (12.5)	42 (18.4)	
ED arrival by EMS									
Yes	33 (20.8)	131 (24.4)	.21	115 (25.8)	49 (19.5)	.09	26 (19.1)	42 (18.4)	.87
No	126 (79.2)	406 (75.6)		330 (74.2)	202 (80.5)		110 (80.9)	186 (81.6)	
Chief signs and symptoms^c^									
Asthma/wheezing	12 (7.4)	54 (10.1)	.48	45 (10.1)	21 (8.4)	<.001	11 (8.1)	20 (8.8)	.10
Cough	16 (10.1)	61 (11.4)		43 (9.7)	34 (13.5)		14 (10.3)	32 (14.0)	
Respiratory (other)	26 (16.4)	82 (15.3)		63 (14.2)	45 (17.9)		22 (16.2)	41 (18.0)	
Seizure	16 (10.1)	50 (9.3)		44 (9.9)	22 (8.8)		14 (10.3)	20 (8.8)	
Fever	12 (7.4)	49 (9.1)		35 (7.9)	26 (10.4)		10 (7.4)	24 (10.5)	
Vomiting	13 (8.2)	41 (7.6)		38 (8.5)	16 (6.4)		12 (8.8)	15 (6.6)	
Altered mental status	18 (11.3)	34 (6.3)		41 (9.2)	11 (4.5)		15 (11.0)	8 (3.5)	
Other	46 (28.9)	166 (30.9)		136 (30.6)	76 (30.3)		38 (27.9)	68 (29.8)	
RePEAT score									
Mean (SD)	1.60 (0.58)	1.57 (0.56)	.60	1.61 (0.55)	1.51 (0.59)	.19	1.59 (0.57)	1.50 (0.58)	.36
Median (IQR)	1.61 (1.13-2.08)	1.54 (1.09-2.02)		1.59 (1.16-2.08)	1.49 (1.00-1.96)		1.59 (1.15-2.03)	1.47 (1.00-1.95)	
RePEAT probability of admission									
Mean (SD)	0.38 (0.23)	0.36 (0.23)	.40	0.38 (0.22)	0.34 (0.23)	.15	0.37 (0.23)	0.33 (0.23)	.27
Median (IQR)	0.36 (0.18-0.56)	0.35 (0.17-0.54)		0.36 (0.19-0.56)	0.31 (0.12-0.52)		0.36 (0.18-0.56)	0.36 (0.12-0.50)	
Distance between ED and UCDCH, km									
Mean (SD)	162.6 (75.5)	151.0 (79.0)	.23	160.3 (79.7)	141.8 (74.6)	.22	162.1 (76.9)	139.4 (74.9)	.19
Median (IQR)	117 (117-240)	117 (80-240)		135 (80-240)	117 (117-240)		126 (99-240)	117 (80-204)	

Abbreviations: ED, emergency department; RePEAT, Revised Pediatric Emergency Assessment Tool; UCDCH, University of California Davis Children’s Hospital.

^a^ From Pearson χ^2^ tests (categorical variables) or Wald tests (continuous variables) adjusted for clustering at the hospital-level.

^b^ Race and ethnicity were combined into a single variable with 4 distinct options: Hispanic of all races, non-Hispanic White, non-Hispanic Black, and non-Hispanic mixed and unreported races. Mixed race includes American Indian or Alaska Native, Asian, Native Hawaiian or other Pacific Islander, and other race or ethnicity.

^c^ Only 7 of the most frequent chief signs and symptoms listed here; other category includes 34 additional chief signs and symptoms, which are factored into the RePEAT score.

As reported in Table 2, 451 patients (84.0%) assigned to the telemedicine arm were transferred to the UCDCH vs 144 patients (90.6%) assigned to the telephone arm. In the intention-to-treat mixed-effects Poisson regression analysis, the adjusted risk of transfer among patients in the telemedicine arm was lower than that of the telephone arm after adjusting for patient age, natural logarithm RePEAT score, and hospital study period (risk rate, 0.93; 95% CI, 0.88-0.99) (Table 3). Among patients transferred to the UCDCH, there was a similar proportion in the telemedicine arm vs the telephone arm admitted to the intensive care unit (75.8% vs 77.1%), ward (7.1% vs 8.3%), and the ED (17.1% vs 14.6%). Of those transferred to the UCDCH ED, 6.5% were discharged home in the telemedicine arm compared with 23.8% in the telephone arm (*P* = .16). The number-needed-to-treat (ie, number needed to provide telemedicine to prevent 1 transfer) was 16.5 (95% CI, 9.1-88.0; *P* = .02).

**Table 2.** Patient Disposition in the Telemedicine and Telephone Arms

Patient disposition	Intention-to-treat analysis			Treatment received analysis			Per-protocol analysis		
	No. (%)		*P* value^a^	No. (%)		*P* value^a^	No. (%)		*P* value^a^
	Assigned telephone (n = 159)	Assigned telemedicine (n = 537)		Received telephone (n = 445)	Received telemedicine (n = 251)		Assigned telephone (n = 136)	Assigned telemedicine (n = 228)	
ED disposition^b^									
Discharged home	7 (4.4)	44 (8.2)	.18	20 (4.5)	31 (12.4)	<.001	4 (2.9)	28 (12.3)	NA
Admitted locally	3 (1.9)	23 (4.3)		4 (0.90)	22 (8.8)		1 (0.7)	20 (8.8)	
Admitted to another hospital	3 (1.9)	17 (3.2)		9 (2.0)	11 (4.4)		3 (2.2)	11 (4.8)	
Transferred to UCDCH^c^	144 (90.6)	451 (84.0)		410 (92.1)	185 (73.7)		127 (93.4)	168 (73.7)	
Died	2 (1.3)	2 (0.4)		2 (0.4)	2 (0.8)		1 (0.8)	1 (0.4)	
Primary outcome: transferred to the UCDCH									
No	15 (9.4)	96 (16.0)	.13	35 (7.9)	66 (26.3)	<.001	9 (6.6)	60 (26.3)	.005
Yes	144 (90.6)	451 (84.0)		410 (92.1)	185 (73.7)		127 (93.4)	168 (73.7)	

Abbreviations: ED, emergency department; NA, not applicable; UCDCH, University of California Davis Children’s Hospital.

^a^ From Pearson χ^2^ tests adjusted for clustering at the hospital level.

^b^ Presenting ED.

^c^ UCDCH PICU, ward, or ED.

**Table 3.** Mixed Effects Poisson Regression Analyses of Transfer to UCDCH Adjusted for Encounter Age, Natural Logarithm RePEAT Score, and Hospital Study Period With ED Random Intercepts

Variable	Intention to treat analysis (n = 692)		Treatment received analysis (n = 692)		Per-protocol analysis (n = 363)	
	RR (95% CI)^a^	*P* value	RR (95% CI)^a^	*P* value	RR (95% CI)^a^	*P* value
Consult modality						
Telephone	1 [Reference]				1 [Reference]	
Telemedicine	0.93 (0.88-0.99)	.02	0.81 (0.71-0.94)	.004	0.79 (0.68-0.92)	.002
Encounter age, y	1.005 (1.0001-1.01)	.045	1.005 (1.0005-1.01)	.03	1.004 (0.996-1.01)	.31
Natural logarithm RePEAT score	1.26 (1.12-1.40)	<.001	1.23 (1.10-1.37)	<.001	1.26 (1.07-1.48)	.006
Hospital study period, 6-mo period						
First	1 [Reference]				1 [Reference]	
Second	1.09 (0.99-1.22)	.09	1.08 (0.99-1.18)	.07	1.10 (0.93-1.31)	.28
Third	1.14 (1.03-1.25)	.01	1.14 (1.05-1.24)	.002	1.25 (1.07-1.47)	.006
Fourth	1.09 (0.95-1.24)	.20	1.11 (0.99-1.24)	.07	1.12 (0.94-1.34)	.21

Abbreviations: ED, emergency department; RePEAT, Revised Pediatric Emergency Assessment Tool; RR, risk rate; UCDCH, University of California Davis Children’s Hospital.

^a^ Risk rates are adjusted RR estimates, with 95% CIs based on robust SEs.

In the treatment-received and per-protocol analyses, we did not find differences in baseline demographic or clinical characteristics between patients in the study arms other than some differences in presenting chief signs and symptoms (Table 1). Similar to the intention-to-treat analysis, we found that patients who received telemedicine consultations in the treatment-received analysis (73.7%) and per-protocol analysis (73.7%) were less likely to be transferred to the UCDCH from the presenting ED than patients who received telephone consultations (treatment received: 92.1%; per protocol: 93.4%). The risk of transfer among patients in the telemedicine arm remained significantly lower in the adjusted treatment-received and per-protocol analyses, as noted in Table 3. The adjusted risk of transfer was significantly lower in the telemedicine arm compared with the telephone arm in both the treatment-received analysis (RR, 0.81; 95% CI, 0.71-0.94) and the per-protocol analysis (RR, 0.79; 95% CI, 0.68-0.92). The number-needed-to-treat with telemedicine was 5.9 (95% CI, 3.6-15.5; *P* = .002) in the treatment-received analysis and 5.1 (95% CI, 3.2-11.8; *P* = .001) in the per-protocol analysis. When the 4 patients who died in the participating ED (2 allocated to the telemedicine arm, 2 allocated to the telephone arm) were excluded from the multivariable analyses and the number-needed-to-treat analyses, the results were substantively unchanged.

Most baseline characteristics were similar between patients who received consultations that adhered to the study arm and those whose consultations deviated from the study arm intervention (eTable in Supplement 2). Consultations for patients who arrived at the presenting ED by emergency medical services were more likely to be conducted by telephone when allocated to telemedicine (67.9%) than for patients who did not arrive by emergency medical services (54.2%). However, mean RePEAT scores were not significantly different between patients whose consultation adhered and did not adhere to study arm assignment. Inclusion of switch period in the multivariable models did not improve the statistical significance of the model fit (*P* = .52 from nested likelihood ratio test).

## Discussion

In this pragmatic, cluster-randomized, crossover study, we compared the influence of telemedicine vs telephone consultations on risk-adjusted transfer of acutely ill children presenting to 15 rural and community EDs in northern California. In the adjusted intention-to-treat analysis, we found that consultations allocated to telemedicine resulted in a significantly lower risk of transfer to a pediatric referral center than those allocated to telephone, which is the current standard of care. These findings were even more dramatic in the treatment-received and the per-protocol analyses. The number-needed-to-treat to reduce 1 transfer was relatively small in all analyses. Our findings support previous research conducted using observational data that the use of telemedicine can support triage transport decisions to potentially reduce secondary overtriage.^10,23,24,25^

Previous studies have reported that telemedicine consultations provided to rural and community EDs have benefits on quality of care, satisfaction, and patient safety.^9,26,27^ To our knowledge, however, the present study is the first randomized clinical trial that demonstrates a decreased transfer rate when telemedicine consultations are encouraged and used compared with telephone consultation.^28^ While interfacility transfers have not been robustly studied, there is some evidence that many transfers may not be needed, are costly for patients and families, and can be associated with an increased risk of adverse events.^5,6,29,30,31^ Our study demonstrates that, by using a relatively low-cost telemedicine intervention, children can be successfully evaluated, treated, and either discharged or admitted locally from their rural and community EDs. Further robust clinical trials are needed to better evaluate the impact of the use of telemedicine in EDs on the patient and family experience, caregiver distress, medication errors, and quality of care.

Our findings are important because they demonstrate that telemedicine may improve local ED clinicians’ ability to care for pediatric patient emergencies. There are current initiatives to increase the pediatric readiness of EDs, especially given evidence that many children live more than 30 minutes away from an ED with a high level of pediatric readiness.^4,32,33^ In addition, investigators have evaluated interfacility transfer patterns and ED capability and found that many hospitals are not fully equipped and may not be providing definitive care to children, resulting in increased transfers to regional pediatric referral centers.^34,35^ Using telemedicine with a remote pediatric specialist provides local physicians and families with more support, both clinically and emotionally, than a standard telephone conversation and could result in lower levels of parental distress.^36,37^ In addition, telemedicine can be used to include the referring bedside nurse, which could increase their confidence in caring for children in their own EDs.^38^

### Limitations

Our study has limitations. As a pragmatic trial, we could not force physicians to use or not use telemedicine or telephone as assigned. We anticipated a high rate of telephone use when encounters were allocated to the telemedicine arm. For these reasons, we used an unbalanced design and conducted an intention-to-treat analysis. In fact, 42.5% of patient encounters allocated to telemedicine actually used telemedicine for the consultation. Patients with consultations that occurred over the telephone when allocated to telemedicine had slightly different chief signs and symptoms and were more likely to have arrived by emergency medical services; however, there did not appear to be clinically significant differences in patient demographic characteristics, patient conditions, or severities of illness for encounters that resulted in protocol deviations. Furthermore, clinical experience suggests that telemedicine and the associated video experience may not necessarily assist in the care or triage decisions in all cases and that consulting physicians may have opted to not use telemedicine when a physical examination was not considered to be helpful in informing their decision-making. Also, because telemedicine consultations generally take longer than telephone consultations, this may have contributed to decisions on which consultation modality was ultimately used. Another limitation was that we did not determine the appropriateness of the transfer or nontransfer by assessing outcomes. While a greater proportion of children in the telephone arm were discharged home following an interfacility transport, suggesting overtriage, it is possible that avoided transfers were inappropriate, particularly in the telemedicine arm. We did not collect outcomes for patients who were discharged from the presenting ED or hospital, and it is therefore possible that some of these children may have had adverse events from not being transferred. However, we believe this is unlikely; no child who was discharged from a participating ED returned to the ED for an issue related to the injury or was admitted or transferred to the UCDCH following a telemedicine or telephone consultation. In addition, this is a study of a telemedicine program that has been in existence for many years and therefore the implementation, use, and impact of other pediatric telemedicine programs may not result in similar findings.

## Conclusions

In this cluster-randomized clinical trial, the use of telemedicine for pediatric critical care consultations to clinicians caring for acutely ill children in rural and community EDs resulted in significantly fewer interfacility transfers. Future studies exploring the benefits of reduced transfer rates on cost, quality of care, medication errors, and patient and family satisfaction are warranted.

## Notes

Supplement 1.
Trial Protocol and Statistical Analysis Plan

Supplement 2. **eTable.** Baseline Characteristics of Nonadherence in the Telemedicine and Telephone Arms

Supplement 3.
Data Sharing Statement

## References

[1] Ames SG, Davis BS, Marin JR, . Emergency department pediatric readiness and mortality in critically ill children. Pediatrics. 2019;144(3):e20190568. doi:10.1542/peds.2019-0568 31444254 PMC6856787

[2] Newgard CD, Lin A, Olson lM, ; Pediatric Readiness Study Group. Evaluation of emergency department pediatric readiness and outcomes among US trauma centers. JAMA Pediatr. 2021;175(9):947-956. doi:10.1001/jamapediatrics.2021.1319 34096991 PMC8185631

[3] Gausche-Hill M, Ely M, Schmuhl P, . A national assessment of pediatric readiness of emergency departments. JAMA Pediatr. 2015;169(6):527-534. doi:10.1001/jamapediatrics.2015.138 25867088

[4] Ray KN, Marin JR, Li J, Davis BS, Kahn JM. Referring hospital characteristics associated with potentially avoidable emergency department transfers. Acad Emerg Med. 2019;26(2):205-216. 30019793 10.1111/acem.13519

[5] Sorensen MJ, von Recklinghausen FM, Fulton G, Burchard KW. Secondary overtriage: the burden of unnecessary interfacility transfers in a rural trauma system. JAMA Surg. 2013;148(8):763-768. doi:10.1001/jamasurg.2013.2132 23784088

[6] Mohr NM, Harland KK, Shane DM, Miller SL, Torner JC. Potentially avoidable pediatric interfacility transfer is a costly burden for rural families: a cohort study. Acad Emerg Med. 2016;23(8):885-894. doi:10.1111/acem.12972 27018337

[7] Brova M, Boggs KM, Zachrison KS, . Pediatric telemedicine use in United States emergency departments. Acad Emerg Med. 2018;25(12):1427-1432. doi:10.1111/acem.13629 30307078 PMC6822676

[8] Zachrison KS, Boggs KM, M Hayden E, Espinola JA, Camargo CA. A national survey of telemedicine use by US emergency departments. J Telemed Telecare. 2020;26(5):278-284. doi:10.1177/1357633X18816112 30558518

[9] Dharmar M, Romano PS, Kuppermann N, . Impact of critical care telemedicine consultations on children in rural emergency departments. Crit Care Med. 2013;41(10):2388-2395. doi:10.1097/CCM.0b013e31828e9824 23921273

[10] Yang NH, Dharmar M, Kuppermann N, . Appropriateness of disposition following telemedicine consultations in rural emergency departments. Pediatr Crit Care Med. 2015;16(3):e59-e64. doi:10.1097/PCC.0000000000000337 25607743 PMC9724605

[11] Dharmar M, Kuppermann N, Romano PS, . Telemedicine consultations and medication errors in rural emergency departments. Pediatrics. 2013;132(6):1090-1097. doi:10.1542/peds.2013-1374 24276844 PMC9724646

[12] Dayal P, Hojman NM, Kissee JL, . Impact of telemedicine on severity of illness and outcomes among children transferred from referring emergency departments to a children’s hospital PICU. Pediatr Crit Care Med. 2016;17(6):516-521. doi:10.1097/PCC.0000000000000761 27099972

[13] Ward MM, Jaana M, Natafgi N. Systematic review of telemedicine applications in emergency rooms. Int J Med Inform. 2015;84(9):601-616. doi:10.1016/j.ijmedinf.2015.05.009 26072326

[14] Schinasi DA, Atabaki SM, Lo MD, Marcin JP, Macy M. Telehealth in pediatric emergency medicine. Curr Probl Pediatr Adolesc Health Care. 2021;51(1):100953. doi:10.1016/j.cppeds.2021.100953 33551336

[15] Baiocchi M, Cheng J, Small DS. Instrumental variable methods for causal inference. Stat Med. 2014;33(13):2297-2340. doi:10.1002/sim.6128 24599889 PMC4201653

[16] Ford I, Norrie J. Pragmatic trials. N Engl J Med. 2016;375(5):454-463. doi:10.1056/NEJMra1510059 27518663

[17] Harris PA, Taylor R, Minor BL, ; REDCap Consortium. The REDCap consortium: building an international community of software platform partners. J Biomed Inform. 2019;95:103208. doi:10.1016/j.jbi.2019.103208 31078660 PMC7254481

[18] Harris PA, Taylor R, Thielke R, Payne J, Gonzalez N, Conde JG. Research electronic data capture (REDCap)—a metadata-driven methodology and workflow process for providing translational research informatics support. J Biomed Inform. 2009;42(2):377-381. doi:10.1016/j.jbi.2008.08.010 18929686 PMC2700030

[19] Gorelick MH, Alpern ER, Alessandrini EA. A system for grouping presenting complaints: the pediatric emergency reason for visit clusters. Acad Emerg Med. 2005;12(8):723-731. doi:10.1197/j.aem.2005.03.530 16079425

[20] National Institutes of health. Racial and ethnic categories and definitions for NIH diversity programs and for other reporting purposes. April 8, 2015. Accessed January 26, 2023. https://grants.nih.gov/grants/guide/notice-files/not-od-15-089.html

[21] Gorelick MH, Alessandrini EA, Cronan K, Shults J. Revised Pediatric Emergency Assessment Tool (RePEAT): a severity index for pediatric emergency care. Acad Emerg Med. 2007;14(4):316-323. doi:10.1197/j.aem.2006.11.015 17312335

[22] Spiegelman D, Hertzmark E. Easy SAS calculations for risk or prevalence ratios and differences. Am J Epidemiol. 2005;162(3):199-200. doi:10.1093/aje/kwi188 15987728

[23] Albritton J, Maddox L, Dalto J, Ridout E, Minton S. The effect of a newborn telehealth program on transfers avoided: a multiple-baseline study. Health Aff (Millwood). 2018;37(12):1990-1996. doi:10.1377/hlthaff.2018.05133 30633672

[24] Haynes SC, Dharmar M, Hill BC, . The impact of telemedicine on transfer rates of newborns at rural community hospitals. Acad Pediatr. 2020;20(5):636-641. doi:10.1016/j.acap.2020.02.013 32081766

[25] Holt T, Sari N, Hansen G, . Remote presence robotic technology reduces need for pediatric interfacility transportation from an isolated northern community. Telemed J E Health. 2018;24(11):927-933. doi:10.1089/tmj.2017.0211 29394155

[26] Dharmar M, Marcin JP, Romano PS, . Quality of care of children in the emergency department: association with hospital setting and physician training. J Pediatr. 2008;153(6):783-789. doi:10.1016/j.jpeds.2008.05.025 18617191 PMC9724612

[27] Marcin JP, Dharmar M, Cho M, . Medication errors among acutely ill and injured children treated in rural emergency departments. Ann Emerg Med. 2007;50(4):361-367, 367.e1-367.e2. doi:10.1016/j.annemergmed.2007.01.020 17433496 PMC9724635

[28] Mitra A, Veerakone R, Li K, Nix T, Hashikawa A, Mahajan P. Telemedicine in paediatric emergency care: A systematic review. J Telemed Telecare. 2021;X211010106. doi:10.1177/1357633X211010106 34590883

[29] Hains IM, Marks A, Georgiou A, Westbrook JI. Non-emergency patient transport: what are the quality and safety issues? a systematic review. Int J Qual Health Care. 2011;23(1):68-75. doi:10.1093/intqhc/mzq076 21123188

[30] Hernandez-Boussard T, Davies S, McDonald K, Wang NE. Interhospital facility transfers in the United States: a nationwide outcomes study. J Patient Saf. 2017;13(4):187-191. doi:10.1097/PTS.0000000000000148 25397857 PMC4956577

[31] Gattu RK, De Fee AS, Lichenstein R, Teshome G. Consideration of cost of care in pediatric emergency transfer-an opportunity for improvement. Pediatr Emerg Care. 2017;33(5):334-338. doi:10.1097/PEC.0000000000000805 27404461

[32] Balmaks R, Whitfill TM, Ziemele B, . Pediatric readiness in the emergency department and its association with patient outcomes in critical care: a prospective cohort study. Pediatr Crit Care Med. 2020;21(5):e213-e220. doi:10.1097/PCC.0000000000002255 32132503

[33] Ray KN, Olson LM, Edgerton EA, . Access to high pediatric-readiness emergency care in the United States. J Pediatr. 2018;194:225-232.e1. doi:10.1016/j.jpeds.2017.10.074 29336799 PMC5826844

[34] Li J, Monuteaux MC, Bachur RG. Interfacility transfers of noncritically ill children to academic pediatric emergency departments. Pediatrics. 2012;130(1):83-92. doi:10.1542/peds.2011-1819 22665410

[35] Whitfill T, Auerbach M, Scherzer DJ, Shi J, Xiang H, Stanley RM. Emergency care for children in the United States: epidemiology and trends over time. J Emerg Med. 2018;55(3):423-434. doi:10.1016/j.jemermed.2018.04.019 29793812

[36] Sauers-Ford HS, Hamline MY, Gosdin MM, . Acceptability, usability, and effectiveness: a qualitative study evaluating a pediatric telemedicine program. Acad Emerg Med. 2019;26(9):1022-1033. doi:10.1111/acem.13763 30974004 PMC6732030

[37] Rosenthal JL, Sauers-Ford HS, Snyder M, . Testing pediatric emergency telemedicine implementation strategies using quality improvement methods. Telemed J E Health. 2021;27(4):459-463. doi:10.1089/tmj.2020.0067 32580661 PMC8060713

[38] Lieng MK, Siefkes HM, Rosenthal JL, . Telemedicine for interfacility nurse handoffs. Pediatr Crit Care Med. 2019;20(9):832-840. doi:10.1097/PCC.0000000000002011 31232857 PMC6726530

