# Supplementary material for: Impact of Tele-Emergency Consultations on Pediatric Interfacility Transfers: A Cluster-Randomized Crossover Trial
Source: JAMA Netw Open. 2023 Feb 13;6(2):e2255770. doi: 10.1001/jamanetworkopen.2022.55770 (PMC9926323; doi:10.1001/jamanetworkopen.2022.55770)
Supplement: Supplement 2. — eTable. Baseline Characteristics of Nonadherence in the Telemedicine and Telephone Arms [file jamanetwopen-e2255770-s002.pdf]

Supplementary Online Content

Marcin JP, Sauers-Ford HS, Mouzoon JL, et al. Impact of tele-emergency consultations on pediatric interfacility transfers: a cluster-randomized crossover trial. *JAMA Netw Open*. 2023;6(2):e2255770. doi:10.1001/jamanetworkopen.2022.55770

eTable. Baseline Characteristics of Nonadherence in the Telemedicine and Telephone Arms

This supplementary material has been provided by the authors to give readers additional information about their work.

© 2023 Marcin JP initial et al. *JAMA Network Open.*

eTable: Baseline characteristics of nonadherence in the telemedicine and telephone arms

Patient factors/Outcome	Telephone Assignment (N=169)			Telemedicine Assignment (N=537)		
	Adhered<br>N=136	Did not Adhere<br>N=23	P*	Adhered<br>N=228	Did not Adhere<br>N=309	P*
Age in years						
Mean (SD)	3.8 (4.5)	3.2 (3.6)	0.51	4.3 (4.4)	4.4 (4.9)	0.79
Median (Q1, Q3)	1.6 (0.3, 6.2)	1.9 (0.2, 5.6)		2.5 (0.7, 7.6)	2.1 (0.3, 8.1)	
Gender, N (%)						
Female	61 (83.7)	12 (16.5)	0.63	92 (39.8)	139 (60.2)	0.25
Male	75 (87.2)	11 (12.8)		136 (44.4)	170 (55.6)	
Insurance status, N (%)						
Private	36 (92.3)	3 (7.7)	0.06	49 (46.7)	56 (53.3)	0.63
Medical/ Self-Pay/No insurance/Other	100 (83.3)	20 (16.7)		179 (41.4)	253 (58.6)	
Race/Ethnicity, N (%)						
Hispanic	19 (95.0)	1 (5.0)	0.27	48 (45.7)	57 (54.3)	0.31
Non-Hispanic White	87 (82.9)	18 (17.1)		123 (42.4)	167 (57.6)	
Non-Hispanic Black	13 (100)	0 (0)		15 (29.4)	36 (70.6)	
Non-Hispanic mixed & other races	17 (81.0)	4 (19.0)		42 (46.2)	49 (53.9)	
ED arrival by emergency medical services, N (%)						
Yes	26 (78.8)	67 (21.2)	0.28	42 (32.1)	89 (67.9)	0.01
No	110 (87.3)	16 (12.7)		186 (45.8)	220 (54.2)	
Chief complaint						
Asthma/Wheezing	11 (8.1)	1 (4.4)	0.004	20 (8.8)	34 (11.0)	0.003
Cough	14 (10.3)	2 (8.7)		32 (14.0)	29 (9.4)	
Respiratory (other)	22 (16.2)	4 (17.4)		41 (18.0)	41 (13.3)	
Seizure	14 (10.3)	2 (8.7)		20 (8.8)	30 (9.7)	
Fever	10 (7.4)	2 (8.7)		24 (10.5)	25 (8.1)	
Vomiting	12 (8.8)	1 (4.4)		15 (6.6)	26 (8.4)	
Altered mental status	15 (11.0)	3 (13.0)		8 (3.5)	26 (8.4)	
Other	38 (27.9)	8 (34.8)		68 (29.8)	98 (31.7)	
RePEAT[#] score						
Mean (SD)	1.59 (0.57)	1.62 (0.69)	0.78	1.50 (0.58)	1.62 (0.54)	0.10
Median (Q1, Q3)	1.59 (1.15, 2.03)	1.74 (0.95, 2.18)		1.47 (1.00, 1.95)	1.60 (1.16, 2.09)	
Driving distance between ED and UCDCH in miles						
Mean (SD)	100.7 (47.8)	103.2 (42.6)	0.91	86.60 (46.57)	99.15 (50.30)	0.11
Median (Q1, Q3)	78 (62, 149)	73 (73, 158)		73 (50, 127)	84 (50, 149)	

© 2023 Marcin JP initial et al. *JAMA Network Open.*

Transferred to UCDCH, N (%)						
No	8 (61.5)	5 (38.5)	0.04	59 (70.2)	25 (29.8)	0.003
Yes	127 (88.2)	17 (11.8)		168 (37.3)	283 (62.8)	
Deceased	1 (50.0)	1 (50.0)		1 (50.0)	1 (50.0)	

*From Chi-square tests (categorical variables) and Wald (continuous variables) adjusted for clustering at the hospital-level

†Revised Pediatric Emergency Assessment Tool (RePEAT)

© 2023 Marcin JP initial et al. *JAMA Network Open.*
