# Supplementary material for: Impact of Tele-Emergency Consultations on Pediatric Interfacility Transfers: A Cluster-Randomized Crossover Trial
Source: JAMA Netw Open. 2023 Feb 13;6(2):e2255770. doi: 10.1001/jamanetworkopen.2022.55770 (PMC9926323; doi:10.1001/jamanetworkopen.2022.55770)
Supplement: Supplement 3. — Data Sharing Statement [file jamanetwopen-e2255770-s003.pdf]

Data Sharing Statement

Marcin. Impact of Tele-Emergency Consultations on Pediatric Interfacility Transfers. *JAMA Netw Open*. Published February 13, 2023. doi:10.1001/jamanetworkopen.2022.55770

Data
Data available: No

Additional Information
Explanation for why data not available: Upon Request
